# Dose-response meta-analysis on urate, gout, and the risk for Parkinson’s disease

**DOI:** 10.1038/s41531-022-00433-5

**Published:** 2022-11-22

**Authors:** Hongtao Chang, Benqiao Wang, Yue Shi, Ruixia Zhu

**Affiliations:** Department of Neurology, The First Affiliated Hospital of China Medical University, Shenyang, China

**Keywords:** Parkinson's disease, Risk factors, Parkinson's disease

## Abstract

The relationship between Parkinson’s disease (PD) and urate or gout has attracted significant interest in recent years, but the results were conflicting. This dose-response meta-analysis aimed to estimate the correlation between urate levels or gout and the risk for PD. The Embase, PubMed, and Medline databases were searched for studies that investigated the relationship between the risk for PD and urate levels or gout. Random-effects or fixed-effects models were used to obtain pooled relative risks (RRs) and corresponding 95% confidence intervals (CIs). Fifteen studies, involving 449,816 participants and 14,687 cases in total, were included in the meta-analysis. High serum urate levels were associated with decreased risk for PD (RR 0.44 [95% CI 0.32–0.55]). Subgroup analysis according to sex revealed a neuroprotective effect of high urate levels against PD among females (0.68 [95% CI 0.43–0.93]) and males (0.49 [95% CI 0.34–0.64]). The risk for PD was lowered by 6% (0.94 [95% CI 0.90–0.98]) for each 1 mg/dl increase in serum urate level and reduced by 13% (0.87 [95% CI 0.80–0.95]) with each 2 mg/dl increase in serum urate level. However, gout was not closely correlated with the risk for PD (0.97 [95% CI 0.85–1.09]). Higher serum urate levels reduced the risk for PD, which was decreased by 6% (relative risk reduction) for each 1 mg/dl increase in serum urate levels. And the results indicated that urate may exert protective effects against the development of PD.

## Introduction

Parkinson’s disease (PD) has become the second most common neurodegenerative disease, and its incidence has increased dramatically in the past decade, imposing heavy health and economic burdens worldwide^1,2^. Because there is currently no effective cure for PD, it is particularly important to investigate the pathogenesis of PD to prevent its onset and progression. Although the pathological mechanisms of degeneration of dopaminergic neurons remain unclear, oxidative stress has been confirmed to play a crucial role^3,4^. Urate is a metabolite of purine and a natural antioxidant that reduces oxidative stress by removing reactive nitrogen and oxygen radicals in vivo and in vitro^5,6^. Therefore, urate may exert potential protective effects against the development of PD. In fact, some epidemiological studies have reported that individuals with hyperuricemia have lower risks for developing PD^7–11^. In contrast, however, other studies reported no significant correlation between high levels of urate and the risk for PD^12,13^.

Gout is a metabolic disorder associated with joint pain caused by elevated urate levels^14^. However, epidemiological studies have reported inconsistent results as to whether gout, such as hyperuricemia, is associated with a lower risk for PD^15–19^. Most previous studies included only single-sex participants and no dose-response analyses, which were not comprehensive and could not demonstrate the strength of the correlation between high urate levels and the risk for PD^13,20^.

Prompted by the conflicting results reported in the literature, a comprehensive meta-analysis of cohort studies was performed to explore the correlation between urate or gout and the risk for PD in both sexes. In addition, a dose-response meta-analysis was also performed to clarify the dose correlation between urate and the risk for PD.

## Results

### Literature search

**Fig. 1:**
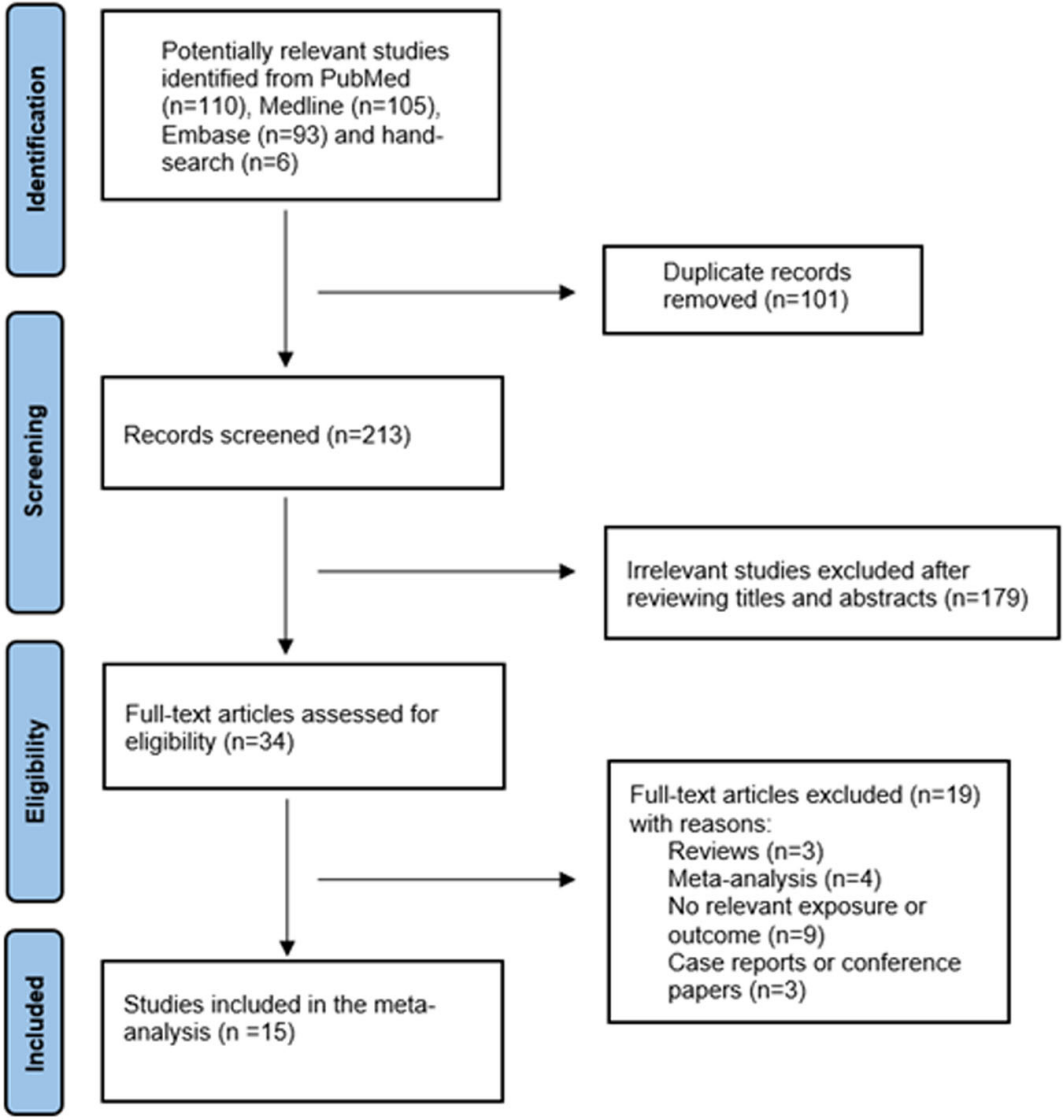
Flow diagram showing the selection process of articles. 15 studies were included in the meta-analysis.

Study selection is illustrated in the flow-diagram presented in Fig. 1. A total of 308 potentially relevant studies were retrieved in the keyword literature search of the computerized databases; an additional 6 were identified through a manual search of the reference lists of these articles. Of 314 studies, 101 were excluded due to duplication, leaving 213 for initial screening of titles and abstracts, among which 179 irrelevant studies were excluded. For the remaining 34 studies, eligibility was accessed through full text reading and 19 did not meet the inclusion criteria. Therefore, 15 studies (all written in English) were ultimately included in this meta-analysis^12,13,16–19,21–29^.

### Characteristics of the included studies

Seven studies investigating gout and the incidence of PD, and eight addressing urate concentration and the risk for PD, involving 449,816 participants and 14,687 cases in total, were identified (Table 1). Six studies were nested case-control studies while the other nine were cohort studies, in which the follow-up ranged from 2 to 24 years. Three studies did not differentiate between the sexes, seven described analyses separately according to sex, two included only males^12,24^, and one described analysis of females only^13^. Participant age ranged from 42.5 to 75.3 years. Aside from sex and age, adjustment for confounders included smoking, alcohol, caffeine, body mass index (BMI) diabetes, and hypertension. The diagnostic criteria for PD, gout, or hyperuricemia were clearly defined in each study. Results of quality assessment according to the NOS (scored from 6 to 9 [mean, 7.5]), indicated that the quality of the included studies was generally good.

**Table 1:** Characteristics of studies on correlation between high urate levels or gout and the risk of PD.

First author, year	Country	Case/total	Gender	Age	Follow-up years	PD definition	Adjustment for confounders	Newcastle-Ottawa scale
Cohort study for gout and PD incidence								
De Vera, 2008^19^	Canada	1182/67457	M/W	74.1	8	The ICD-9 code	Age, sex, hypertension, diabetes, hyperlipidemia, COPD, Charlson comorbidity score, use of diuretics, and use of NSAIDs	Selection: 4 stars Comparability: 2 stars Outcome: 3 stars
Pakpoor, 2015^22^	England	1568/217179	Combined	69.4	5	The ICD 9 and 10 revision codes	Age, sex, calendar year of admission, region of residence, and socioeconomic status	Selection: 3 stars Comparability: 1 star Outcome: 2 stars
Singh, 2019^21^	US	1129/21507	Combined	75.3	2.38	ICD-9-CM diagnostic code	Age, sex, hypertension, hyperlipidemia, and statin use	Selection: 3 stars Comparability: 2 stars Outcome: 3 stars
Hu, 2020^17^	China	339/7900	M/W	50	13.36	A code: A221; ICD-9-CM code: 332	Sex, age, urbanization, typical comorbidities, and monthly income	Selection: 3 stars Comparability: 2 stars Outcome: 2 stars
Case-control study for gout and PD incidence								
Alonso, 2007^18^	UK	1052/6634	M/W	70.0/68.7	6	Computer-recorded PD with confirmation of the related paper records	Age, sex, practice, year of enrollment in the GPRD, and smoking	Selection: 3 stars Comparability: 2 stars Outcome: 2 stars
Schernhammer, 2013^16^	Denmark	4484/22416	M/W	–	7	Diagnostic code and prescription of PD medication	Age, sex, and COPD	Selection: 3 stars Comparability: 1 star Outcome: 2 stars
Lai, 2014^23^	China	3854/15416	M/W	75.0/74.0	10	ICD-9	Sex, age, diabetes, stroke, dementia, hyperlipidemia, depression, renal failure, head injury, hypertension, and polypharmacy	Selection: 3 stars Comparability: 1 star Outcome: 2 stars
Cohort study for urate and PD incidence								
Weisskopf, 2007^24^	US	84/165	M	30–55	9	Diagnostic validations for self-reported PD by treating neurologists or internists	Age, smoking, and caffeine intake	Selection: 3 stars Comparability: 2 stars Outcome: 2 stars
de Lau, 2005^25^	Netherlands	68/4695	Combined	55–	9.4	Screened positive received a structural diagnostic workup	Age, sex, smoking, alcohol, dairy products, BMI	Selection: 3 stars Comparability: 2 stars Outcome: 3 stars
Davis, 1996^12^	US	92/6851	M	45–68	24	By neurologists or neurosurgeons	Age, smoking	Selection: 3 stars Comparability: 1 star Outcome: 2 stars
Chen, 2009^27^	US	95/15036	M/W	45–64	17	ICD diagnosis or self-reported PD with confirmation by movement disorder specialist	Age, sex, race, smoking, caffeine, BMI, alcohol, serum creatinine	Selection: 3 stars Comparability: 2 stars Outcome: 3 stars
Jain, 2011^28^	US	154/5749	M/W	67–78	14	Self-report, medication, and hospitalization records	Age, smoking, race, BMI, alcohol, creatinine, diabetes, hypertension, uricosurics, EKG	Selection: 4 stars Comparability: 2 stars Outcome: 3 stars
Case-control study for urate and PD incidence								
O’Reilly, 2010^13^	US	101/504	W	68	14	Self-reported PD with confirmation by treating physicians and movement disorder specialist	Age, smoking, and caffeine	Selection: 3 stars Comparability: 2 stars Outcome: 3 stars
Winquist, 2010^26^	US	97/57040	M/W	18–	2	Self-reported PD and reported current use of a standard PD medication	Age, gender, smoking, BMI, alcohol	Selection: 4 stars Comparability: 2 stars Outcome: 3 stars
Gao, 2016^29^	US	388/1267	M/W	30–79	24	By treating neurologist/internists or movement disorder specialist	Age, smoking status, height, weight, presence of chronic diseases, and consumption of caffeinated coffee and alcohol, BMI	Selection: 3 stars Comparability: 2 stars Outcome: 3 stars

### Serum urate levels and the risk for PD

#### Pooled RR

**Fig. 2:**
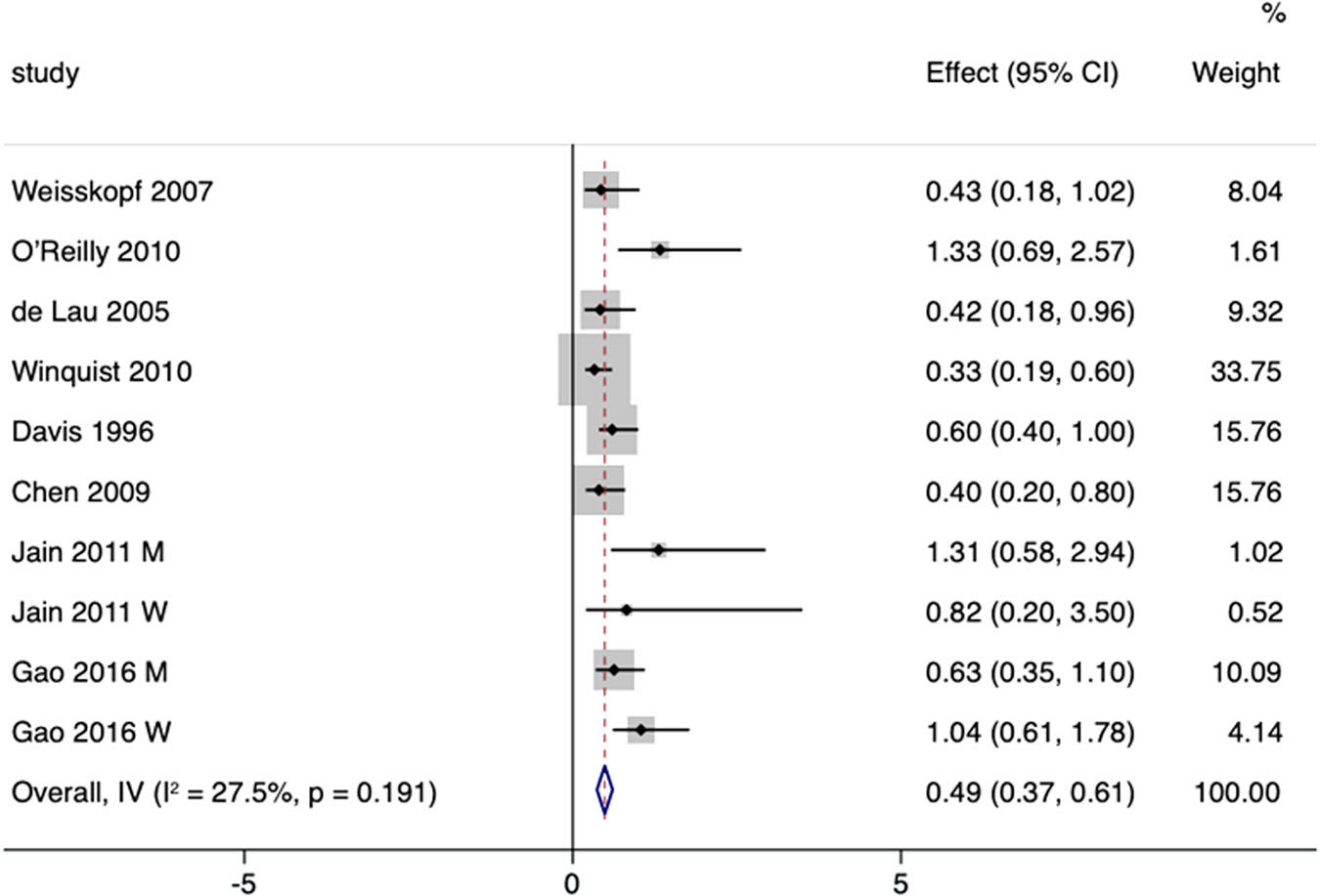
RRs with 95% CIs from studies on the correlation between high urate concentration and risks of PD. The adjusted pooled estimate was 0.44 (95% CI 0.32–0.55).

**Fig. 3:**
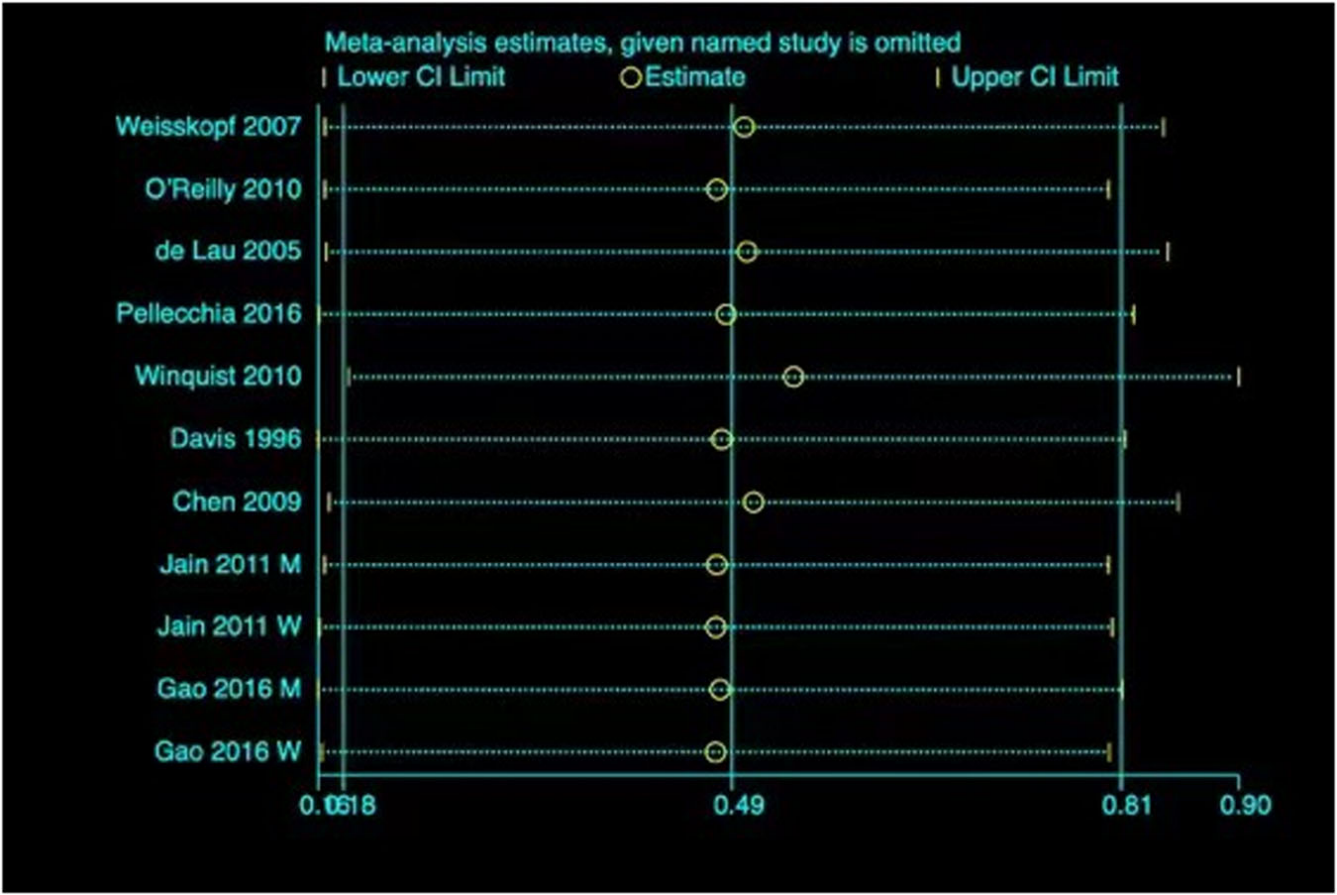
Sensitivity analysis omitting one article in turn from the analyses on the correlation between high urate concentration and PD risks. The vertical lines show the summary estimate (0.49) and its 95% CI (0.18–0.81) from the meta-analysis of all the articles.

Eight studies including 91,307 participants and 1079 cases were included to assess the relationship between urate concentration and the risk for PD^12,13,24–29^. The pooled estimate using fixed-effects model was 0.49 (95% CI 0.37–0.61) with low heterogeneity (*I*² = 27.5%; *p* = 0.191) (Fig. 2). Begg’s test and funnel plot revealed no substantial publication bias (*p* = 0.152), while Egger’s test revealed publication bias (*p* = 0.008). The trim-and-fill method was applied, and the adjusted estimate was 0.44 (95% CI 0.32–0.55), indicating that high serum urate levels decreased the risk for PD. In sensitivity analysis, removing each study in turn did not substantially alter the pooled estimate (Fig. 3).

#### Subgroup analysis

**Fig. 4:**
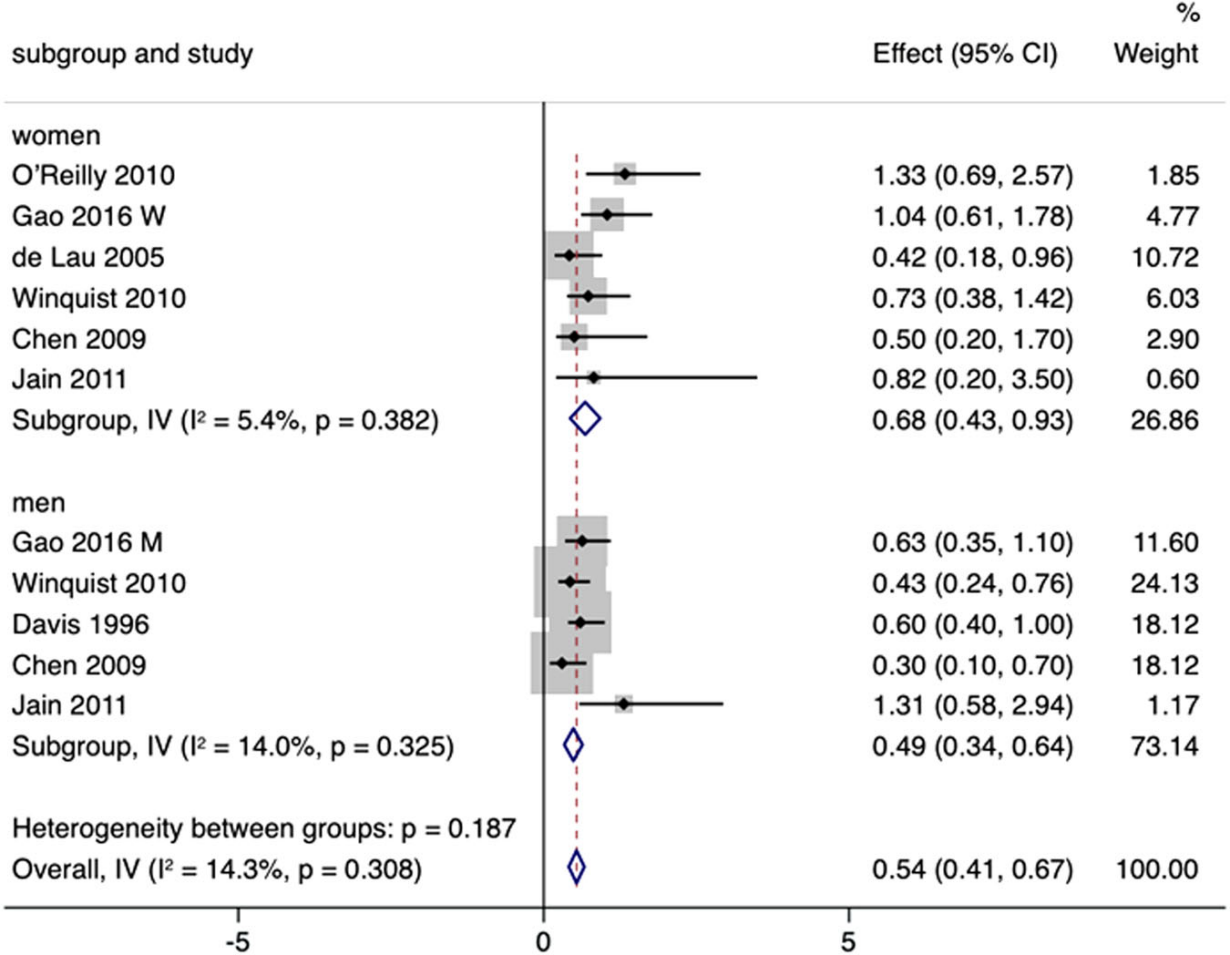
Subgroup analyses by sex on the correlation between urate concentration and PD risk. RRs were 0.68 (95% CI 0.43–0.93) in females and 0.49 (95% CI 0.34–0.64) in males.

Subgroup analysis according to sex was performed to investigate whether high urate levels reduced the risk for PD differently for males and females (Fig. 4). Five studies were included for analyses of females and six for males. Compared with overall (RR 0.54 [95% CI 0.41–0.67]), the neuroprotective effect of high serum urate levels against PD existed in both females (RR 0.68 [95% CI 0.43–0.93]) and males (RR 0.49 [95% CI 0.34–0.64]), without significant heterogeneity (*p* = 0.187). Begg’s test and funnel plot revealed no substantial publication bias (*p* = 0.101), whereas Egger’s test revealed publication bias (*p* = 0.018), and the trim-and-fill method was applied.

#### Dose-response analysis

**Fig. 5:**
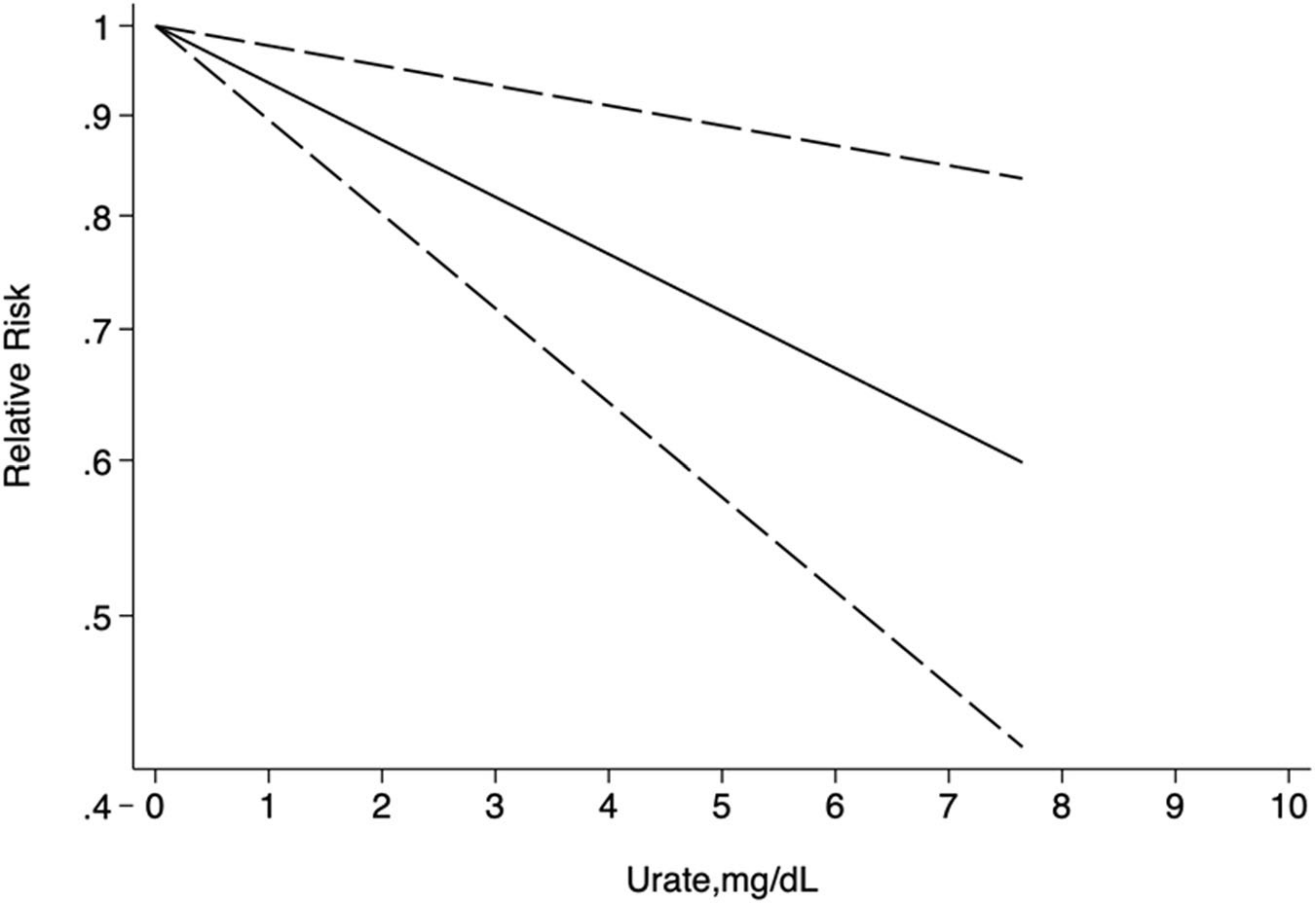
Dose–response correlation between urate levels and PD risk. Restricted cubic spline revealed a negative linear correlation.

Data from five studies were used in the linear dose-response analysis of urate concentration and the risk for PD^12,13,25,26,29^. The nonlinear correlation between urate levels and the risk for PD was not significant (*p* = 0.209). Restricted cubic spline was used to model the linear dose-response correlation, which revealed a negative linear correlation between urate levels and the risk for PD (Fig. 5). The risk for PD was lowered by 6% (RR 0.94 [95% CI 0.90–0.98]) for each 1 mg/dl increase in urate level. Moreover, PD risk was further decreased by 13% (RR 0.87 [95% CI 0.80–0.95]) with each 2 mg/dl increase in serum urate level.

#### Gout and the risk for PD

**Fig. 6:**
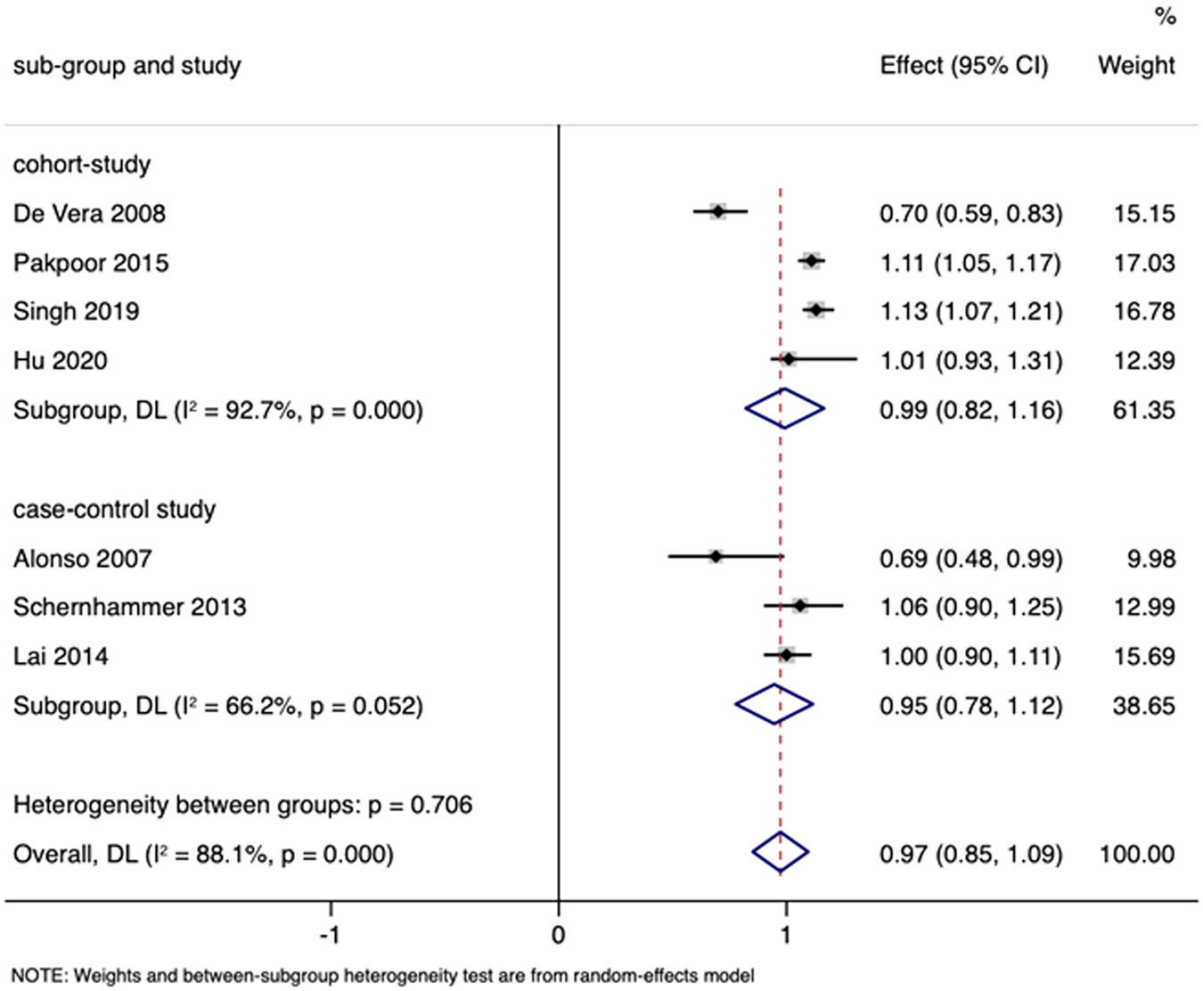
RRs with 95% CIs from studies on the correlation between gout and PD risk. The pooled estimate was 0.97 (95% CI 0.85–1.09).

**Fig. 7:**
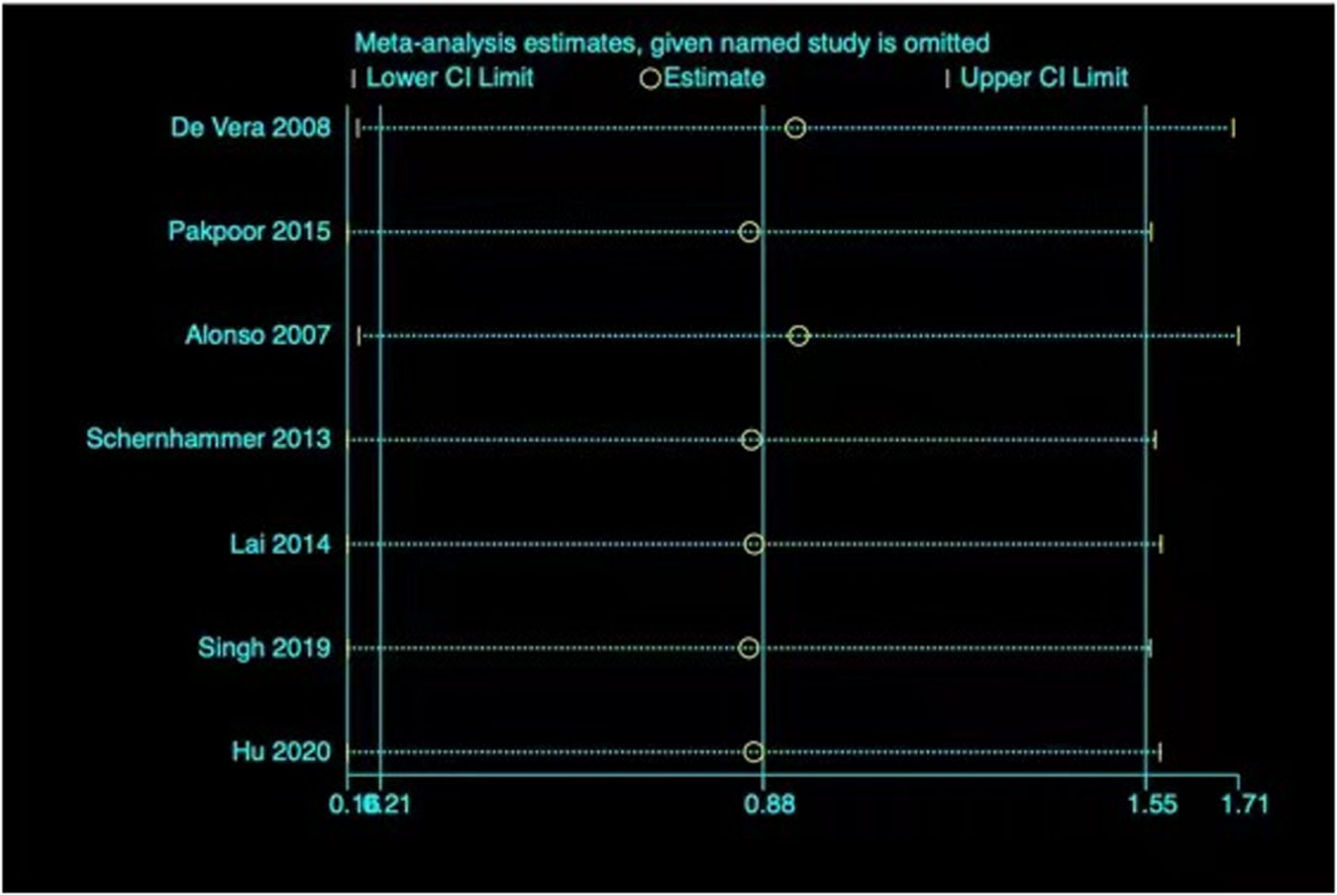
Sensitivity analysis omitting each study in turn from the analyses on the correlation between gout and the risk of PD. The vertical lines show the summary estimate (0.88) and its 95% CI (0.21–1.55) from the meta-analysis of all the articles.

Four cohort studies and three nested case-control studies involving 358,509 participants and 13,608 cases were adopted into analyses of gout and the risk for PD^16–19,21–23^. The pooled RR in the random-effects model was 0.97 (95% CI 0.85–1.09) with high heterogeneity (*I*² = 88.1%; *p* = 0.000), indicating that gout was not closely correlated with the risk for PD (Fig. 6). Furthermore, the subgroup results of nested case-control studies (RR 0.95 [95% CI 0.78–1.12]) and cohort studies (RR 0.99 [95% CI 0.82–1.16]) were consistent with the overall result. No significant publication bias was evident according to Begg’s (*p* = 0.230) and Egger’s (*p* = 0.133) tests. Furthermore, the funnel plot displayed symmetry. The pooled estimates were substantially altered after excluding one study at a time (Fig. 7).

**Fig. 8:**
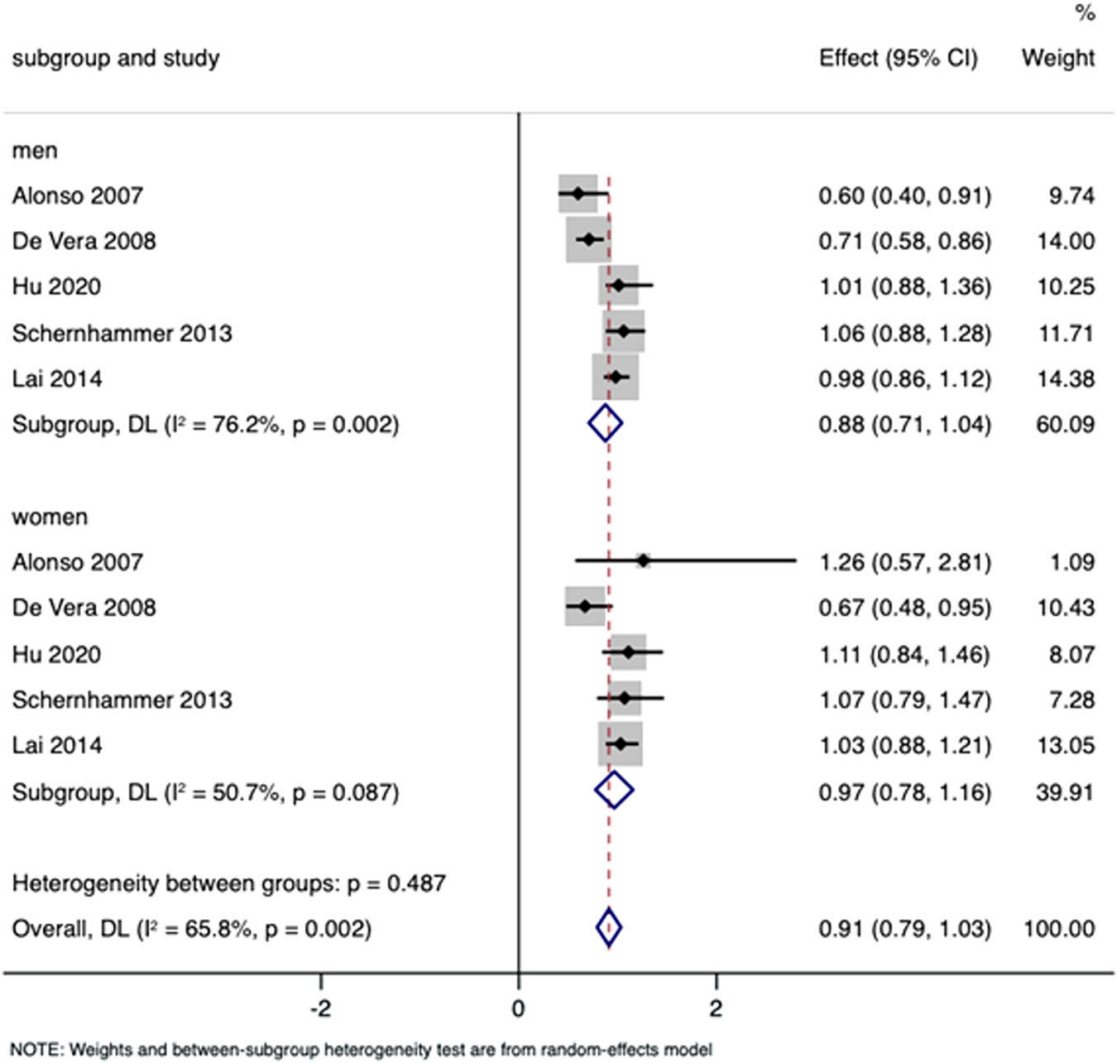
Subgroup analyses by sex on the association between gout and PD risk. RRs were 0.97 (95% CI 0.78–1.16) in females and 0.88 (95% CI 0.71–1.04) in males.

Five studies were included for subgroup analysis according to sex to explore whether gout was related to the risk for PD for only males or only females^16–19,23^. The pooled estimate for females was 0.97 (95% CI 0.78–1.16) and the pooled RR for males was 0.88 (95% CI 0.71–1.04), without substantial heterogeneity (*p* = 0.487) (Fig. 8). Publication bias was not evident according to Begg’s test (*p* = 0.721), Egger’s test (*p* = 0.682), and symmetrical funnel plots. The results do not suggest a strong relationship between gout and the risk for PD.

## Discussion

The relationship between PD and urate or gout has attracted significant interest in recent years. However, published studies have reported conflicting results regarding these associations. A study by Jain^28^ reported that high urate levels were not associated with the reduction in risk for PD, while a meta-analysis by Shen^10^ indicated a 33% decrease in the risk for PD among participants with higher urate concentrations. Regarding the correlation between gout and risk for PD, a study by Alonso^30^ indicated that the risk for PD was lower among individuals with gout, whereas the study by Ungprasert^15^ reported that gout was not correlated with a lower risk for PD. To address the controversies in the existing literature, meta-analysis was the most reliable way to combine all the information.

Accordingly, we performed a meta-analysis of all eligible data including 449,816 participants and 14,687 cases from 15 studies. With the very large sample size, high quality of the studies, and high stability of the sensitivity analysis, results obtained in our meta-analysis were highly convincing. Our study found that high urate levels reduced the risk for PD. In the dose-response analysis, the risk for PD was reduced by 6% (relative risk reduction) for each 1 mg/dl increase in serum urate level. For each 2 mg/dl increase in serum urate level, the risk for PD was decreased by 13% (relative risk reduction).

In the analysis of gout and risk for PD, results revealed no strong correlation. Although gout is a metabolic disorder caused by prolonged hyperuricemia, the effect of gout on the risk for PD was not the same as the influence of high serum urate levels. A possible explanation is that patients with gout are likely to take medication that lowers the concentration of serum urate or undergo anti-inflammatory therapy, which may influence the risk for PD. Chen et al. conducted a meta-analysis to explore non-genetic risk factors for PD, and indicated that among non-steroidal anti-inflammatory drugs, aspirin intake increased the risk of PD (RR 1.12 [95% CI 1.01–1.23])^31^.

We also performed subgroup analysis of urate level according to sex and found that high serum urate levels reduced the risk for PD in both males and females. A study by Shen^10^ indicated that the neuroprotective effect of serum urate was evident in only males and not females. However, our study incorporated more comprehensive data and revealed that the neuroprotective effects of high urate levels also exist in females.

Oxidative damage, including lipid peroxidation and DNA damage, was evident in autopsy of brains with PD, suggesting that oxidative stress plays a vital role in the mechanisms of PD^32^. There are several hypotheses for why high urate levels lower the risk for PD. First, as the anion of uric acid, urate is a key antioxidant that can reduce oxidative damage through removing reactive oxygen species (ROS) and free radicals. Second, urate can chelate metal ions and reduce their ability of oxidize^33^. In an animal study, urate reduced oxidative stress in dopaminergic cells induced by iron^34^. Third, in addition to the reduction in ROS and radicals, potential mechanisms for the protective effect for neurons of urate include the maintenance of mitochondrial function and protection from apoptosis induced by dopamine. A previous study found the metabolism of dopamine causes oxidative damage in substantia nigra, which can be reduced by urate^35^.

Previous meta-analyses exploring the correlation between urate or gout and the risk for PD included a small number of studies and drew different conclusions. The studies by Shen and Gao indicated that males—but not females—with high serum urate levels had lower risks for PD^10,29^. The study by Alonso indicated that the risk for PD was lower among participants with gout, while the study by Ungprasert reported that gout was not associated with lower risks for PD^15,18^. Our study included more high-quality studies and we performed subgroup analyses. We found that both males and females with high urate levels exhibited lower risks for PD. We also performed dose-response analyses to illustrate the extent to which high serum urate concentrations reduce the risk for PD more precisely.

The present meta-analysis had some strengths. First, we searched the Embase, PubMed, and Medline databases and included 15 prospective studies with a very large number of participants from different countries, which enabled us to comprehensively identify correlations and reduce potential sample error. Second, the studies we included were of high quality according to the NOS, which meant that results of our meta-analysis are highly reliable. Third, we performed a dose-response analysis and generated linear dose-response curves, which provided a quantitative estimation and an intuitive plot of the correlation between high urate levels and the risk for PD. Fourth, compared to previous single-sex or mixed-sex studies, we performed separate subgroup analyses according to sex and found the neuroprotective effect of serum urate exists among both males and females.

However, there were limitations to our study. First, although all the included studies controlled for confounding factors, the adjusted confounders were different in each study. Because there is still much to explore about PD, there may be residual confounders that have an impact on the results. Second, we could not exclude the presence of publication bias in this study, although Begg’s test revealed little publication bias and we applied the trim-and-fill method. Third, heterogeneity was high in the gout analysis, which may have influenced the interpretation of the results. Fourth, the number of studies investigating the correlation between urate or gout and the risk for PD is not large, and only part of the studies was included due to the rigorous inclusion criteria of our meta-analysis.

The neuroprotective effect of high urate levels for PD has potentially important implications for prevention. The concentration of urate can be increased by intake of a purine-rich diet or administration of inosine^36^, which is the precursor of purine and has already been used for the clinical treatment of multiple sclerosis^37^. Schwarzschild et al. conducted the SURE-PD3 randomized clinical trial, and identified that treatment with inosine did not lead to a significant difference compared with placebo in the disease progression among patients recently diagnosed with PD^38^. The results did not recognize inosine as a treatment for early PD. However, Shen’s study reported that the progression of PD was slow among patients with high serum urate levels^10^. Future studies with larger sample sizes are needed to further clarify the link between adenosine or high urate concentrations and PD progression. Besides, high serum urate level is a risk factor for hypertension, stroke, cardiovascular and metabolic diseases, such as diabetes mellitus^39–42^, which means it is important to weigh the benefits of lowering the risk for PD and adverse effects and maintain the concentration of serum urate in a suitable range. In future studies, the effect of urate on the risk for PD will be further confirmed, and a potentially new avenue will be provided for the treatment of PD.

In conclusion, results of the present meta-analysis indicated that high serum urate levels reduced the risk for PD and, in the dose-response analysis, the risk for PD was reduced by 6% (relative risk reduction) for each 1 mg/dl increment in serum urate level. Our results indicated that urate may exert protective effects against the development of PD. And gout did not have a significant correlation with a lower risk of PD. More detailed studies investigating the effects of high urate levels or gout on PD are needed to warrant.

## Methods

### Literature search strategy

This study is reported in accordance with the Preferred Reporting Items for Systematic Reviews and Meta-Analyses (i.e., PRISMA) and Meta-Analysis of Observational Studies in Epidemiology (i.e., MOOSE) guidelines^43,44^. The PubMed, Medline, and Embase databases were searched for relevant studies published before March 2022, without language or country restrictions. The MeSH terms “urate”, “uric acid” or “hyperuricemia”, “gout”, “cohort”, “prospective” “incidence”, “risk” “follow up” and “Parkinson’s disease” were used in the literature search. Boolean operators were used in this way: (“urate” OR “uric acid” OR “hyperuricemia”) AND “Parkinson’s disease” AND (“cohort” OR “prospective” OR “incidence” OR “risk” OR “follow up”). The reference lists of the retrieved articles and correlative reviews were also manually searched for other potentially eligible studies.

### Selection criteria

To be included in the meta-analysis, studies must have fulfilled the following criteria: reported diagnostic criteria for PD; hyperuricemia and gout clearly stated; published in peer-reviewed journals; cohort or case-control studies assessing the correlation between urate or gout and the risk for PD; and relative risks (RRs) or odds ratios (ORs) with corresponding 95% confidence intervals (CIs) could be obtained. For the dose-response analysis, the concentrations of serum urate were required and divided into ≥ 3 groups of different levels.

The quality of the included studies was evaluated according to the Newcastle-Ottawa Scale (NOS) in terms of selection, comparability, and outcome^45^. The NOS scores nine stars at most, and studies scoring no less than six stars were considered to be of high-quality.

### Data extraction

The following data were independently extracted from the articles by two reviewers using a standardized data form: first author’s last name; publication year; region of study; study population; mean age; sex distribution; follow-up; number of cases; definition of outcome; adjusted confounders; serum levels of urate; and corresponding risk estimates and 95% CI. The result adjusted by the most confounders was chosen in the study with several adjusted models. Disagreements were resolved by consensus discussion.

### Statistical analysis

Some studies used international units (μmol/liter) to report uric acid levels, which were converted to traditional units (mg/dl) using 1 mg/dl = 59.48 μmol/l. RRs with corresponding 95% CIs were used to explore the correlation between urate level or gout and the risk for PD. The lowest level of serum urate was regarded to be the reference value, and serum levels of urate in each group are expressed as mean concentration. If a level group had upper and lower bounds, the average value of both bounds was calculated as the mean concentration of the group. For the group without a lower or upper bound, the mean concentration was the upper or lower boundary plus or minus one-half the range of the closest group.

Data analysis was performed using Stata version 15.1 (Stata Corp LLC, College Station, TX, USA) and differences with a two-sided *P* < 0.05 were considered to be statistically significant. Heterogeneity was evaluated using the *I*² statistic and Cochran’s Q statistic^46^. An *I*² < 50% suggested no obvious heterogeneity and the adjusted RR was pooled using the fixed-effects model. Otherwise, pooled estimates based on the random-effects model under the condition that substantial heterogeneity existed (i.e., *I*² > 50%)^47^. Subgroup analysis was performed according to sex. Begg’s test and Egger’s test were performed to assess publication bias, in which *p* < 0.05 indicated statistically significant publication bias^48,49^. Asymmetrical funnel plots also suggested potential publication bias. When publication bias was indicated, the pooled estimate was adjusted using the “trim-and-fill” method^50^. Sensitivity analysis was performed to evaluate the stability of the result by removing one study at a time and calculating the pooled RRs of the other studies.

Generalized least squares regression was used to estimate the dose-response correlation between urate levels and the risk for PD, considering covariance for each exposure category among the different studies^51,52^. First, a linear association was assumed. RR estimates were calculated per mg/dl serum urate concentration increase and then pooled. Second, the restricted cubic spline model with three knots was applied to check for possible non-linear associations. The *P* values for non-linearity were obtained by testing the null hypothesis, for which the second spline’s coefficient was zero.

### Reporting summary

Further information on research design is available in the Nature Research Reporting Summary linked to this article.

## Supplementary information

Reporting Summary

## Data Availability

The data used in this paper have been previously published, and details are reported in Table 1.
